# Patient safety incidents in Irish general practice during the COVID-19 pandemic: an exploratory practice level analysis

**DOI:** 10.1186/s12875-024-02439-9

**Published:** 2024-05-29

**Authors:** Nathaly Garzón-Orjuela, Claire Collins, Sara Willems, Esther Van Poel, Akke Vellinga

**Affiliations:** 1 School of Public Health, Physiotherapy and Sports Science, University College Dublin, Dublin, Ireland; 2 CARA Network, School of Public Health, Physiotherapy and Sports Science, University College Dublin, Dublin, Ireland; 3 Irish College of General Practitioners, Dublin, Ireland; 4 Department of Public Health and Primary Care, Ghent University, Ghent, 9000 Belgium

**Keywords:** Patient safety, COVID-19, Infectious diseases, General practice, Adverse events, Incidents, Medical errors

## Abstract

**Background:**

Patient safety is defined as the prevention of harm to patients and aims to prevent errors. This analysis explores factors associated with the reported occurrence of patient safety incidents (PSIs) in general practices in Ireland at the start of the COVID-19 pandemic.

**Methods:**

The PRICOV-19 was a cross-sectional study to record the (re)organisation of care provided in general practice and changes implemented during the COVID-19 pandemic in 38 countries. Primary outcomes include three potential scenarios of PSIs: delayed care due to practice factors, delayed care due to patient factors, and delayed care due to triage. Exploratory variables included demographic and organisational characteristics, triage, collaboration, and strategies to safeguard staff members’ well-being.

**Results:**

Of the 172 participating Irish general practices, 71% (*n* = 122) recorded at least one potential PSI. The most frequent incident was delayed care due to patient factors (65%), followed by practice (33%) and triage (30%). Multivariate analysis showed that delayed care due to patient factors was associated with changes in the process of repeat prescriptions (OR 6.7 [CI 95% 2.5 to 19.6]). Delayed care due to practice factors was associated with suburbs/small towns (OR 4.2 [1.1 to 19.8]) and structural changes to the reception (OR 3.5 [1.2 to 11.4]). While delayed care due to patient factors was associated with having a practice population of 6000–7999 patients (OR 4.7 [1.1 to 27.6]) and delayed care due to practice factors was associated with having a practice population of 2000–3999 patients (OR 4.2 [1.2 to 17.1]). No linear associations were observed with higher or lower patient numbers for any factor. Delayed care due to triage was not associated with any exploratory variables.

**Conclusion:**

The COVID-19 pandemic resulted in dramatic changes in the delivery of care through general practices in Ireland. Few factors were associated with the reported occurrence of PSIs, and these did not show consistent patterns. Sustained improvements were made in relation to repeat prescriptions. The lack of consistent patterns, potentially confirms that the autonomous decisions made in general practice in response to the challenges of the COVID-19 pandemic could have benefitted patient safety (See Graphical abstract).

**Graphical Abstract:**

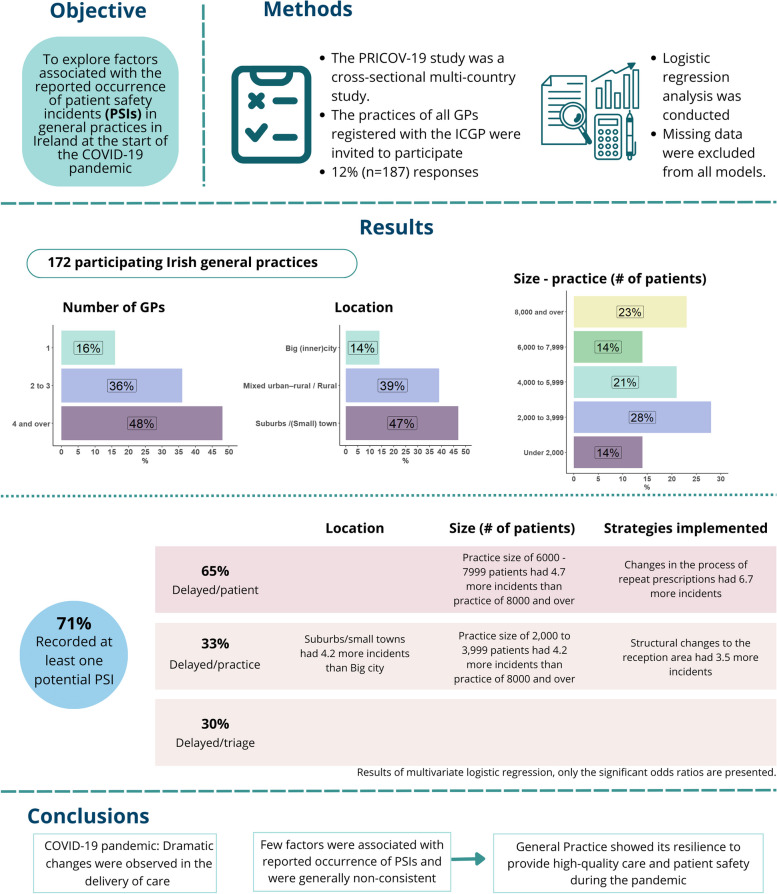

**Supplementary Information:**

The online version contains supplementary material available at 10.1186/s12875-024-02439-9.

## Background

Patient safety is related to reducing and preventing risks, adverse events, and incidents associated with health care provision [1]. The Royal College of General Practitioners defined patient safety incidents (PSI) in general practice as “*an unexpected event which could have, or did, lead to harm for one or more patients receiving healthcare*” and it is crucial to inform and investigate the incident even if it does not result in harm to prevent similar future incidents [2]. PSIs might occur during any healthcare process relating to access, prescription, diagnosis, treatment, or communication among health professionals (inter-professionals) and patients [3]. In 2018, 20–25% of primary care patients reported at least one incident in developing countries of which 80% were preventable [4, 5]. Up to a quarter of the general population experienced an unintended event in primary care, most of which were in relation to diagnosis or prescribed medications [4, 5].

COVID-19 was declared an international public health emergency in February 2020 [6], putting an immediate strain on health systems [7]. Sudden changes involved patient consultations, public health performance responsibilities, and communication [7, 8]. However, strategies implemented in primary care to cope during the COVID-19 pandemic have resulted in issues for non-COVID-19 patients, such as delays in diagnosis, assessments, referrals and treatments, and complications deriving from isolation at home [9, 10].

In Ireland, Curran et al. analysed contributing factors to PSIs of general practitioners (GPs) in 2017. They identified the situational factors domain as the most common contributing factors in all GPs, which includes service user (patient characteristics), task characteristics, individual staff, and team factors [11]. In response to the COVID-19 pandemic, general practices implemented telemedicine consultations and often reduced (non-COVID-19-related) consultations, and it is critical to understand the impact of these changes [12]. Therefore, this study aimed to explore factors associated with the reported occurrence of PSIs in general practices in Ireland at the start of the COVID-19 pandemic.

## Methods

The PRICOV-19 study was a cross-sectional multi-country study with the aim to record the (re) organisation of care provided by general practice and changes implemented due to the pandemic. Details of this cross-sectional study and methods have been described in detail elsewhere [13–16]. This study focuses on the results from general practices in Ireland. The practices of all GPs registered with the Irish College of General Practitioners (ICGP) were invited to participate and responses were obtained from 12.2% (*n* = 187); completing one online self-reported questionnaire per practice [13] from January to March 2021.

### Outcomes

The primary outcome of this analysis was collected through five questions which focused on general practice since the start of the COVID-19 pandemic:

- a patient with a fever caused by an infection other than COVID-19 was seen late due to the fact the COVID-19 protocol was followed which delayed the care,
- a patient with an urgent condition was seen late because he/she did not come to the practice sooner,
- a patient with a serious condition was seen late because he/she did not know how to call on a GP,
- a patient with an urgent condition was seen late because the situation was assessed as non-urgent during the telephone triage, and.
- a patient with an urgent condition other than COVID-19 was assessed incorrectly during the triage procedure.

These were categorised into three potential patient safety outcomes:

1. Delayed care due to practice factors (yes to question a),
2. Delayed care due to patient factors (yes to question b or c), and.
3. Delayed care due to triage (yes to question d or e).

### Exploratory factors

The exploratory variables were related to demographic and organisational characteristics of practices (location, number of staff and role, and size of the practice) and strategies implemented since COVID-19 (triage, collaboration, and measures guarding patient safety). Explanatory factor selection was based on a potential relationship with PSIs reported in the literature (patient knowledge, organisational, environmental, care procedures, and care coordination) [10, 17, 18].

### Statistical analysis

Descriptive univariate logistic regression was used to assess the individual associations between primary outcomes and exploratory variables. Multivariable logistic regression analysis was conducted to evaluate the association between primary outcomes and exploratory factors, excluding multicollinear and confounding variables. Multicollinearity was checked through calculating the variance inflation factor (VIF), and exploratory variables with a VIF less than 5 (low correlation) remained in the models. Confounding was assessed using change-in-estimate criterion (cut-off < 30%) [19]. Missing data were excluded from all models. Associations were reported in odds ratios (OR) with 95% confidence intervals (CI). The analysis was carried out in R software (version 4.1.0).

## Results

Overall, 172 general practices (92%) completed at least one of the five PSI questions and were included in this analysis. Table 1 presents the characteristics of the general practices and strategies implemented during the COVID-19 pandemic. No significant differences were observed between included practices and practices with missing PSI data. Most practices were located in suburbs or small towns (47%), with four or more GPs (48%) and more than 4,000 registered patients (58%). If telephone triage was done by someone other than a GP, 92% could always rely on immediate support from a GP, and 69% of practices had online team meetings to discuss incidents about quality of care. Triage before consultation, increasing infection control practices, and video consultations were strategies used by practices to safeguard the well-being of the staff members.

**Table 1:** Main characteristics of general practices and strategies implemented during the COVID-19 pandemic (*n* = 172)

Characteristics of practices	*n*	%
**Location of practice**		
Big (inner)city	24	14.1
Suburbs /(Small) town	79	46.5
Mixed urban–rural / Rural	67	39.4
**Number of people actively work**		
Paid staff		
1 to 10	96	56.8
11 to 20	65	38.5
20 and over	8	4.7
Unpaid staff		
1	16	11.0
2	5	3.4
3	1	0.7
**Number of GPs**		
1	27	15.9
2 to 3	62	36.5
4 and over	81	47.6
**Number of GP trainees**		
0	95	57.9
1	60	36.6
2	9	5.5
**Staff working in practice**		
Practice manager	68	39.5
Nurse or care providers	148	86.0
**Size of this practice (number of patients)**		
Under 2,000	24	14.3
2,000 to 3,999	47	28.0
4,000 to 5,999	35	20.8
6,000 to 7,999	23	13.7
8,000 and over	39	23.2
**Strategies implemented**		
In the situation where telephone triage is performed by someone other than a GP and he/she needs support when assessing a call, he/she can rely on support from a GP.		
Always	142	91.6
Never/Rarely/Sometimes/Mostly	13	8.4
If an incident about quality of care occurs, this is discussed at a(n) (online) team meeting (either with the whole team or only with the health professionals)		
Always	108	68.8
Never/Rarely/Sometimes/Usually	49	31.2
Measures guarding patient safety		
Performing triage before patients enter this practice	161	98.2
Limiting the number of patients in the waiting room	136	82.9
No longer use the waiting room	62	37.8
Telephone triage	158	96.3
Structural changes to the reception area	123	75.0
Increasing infection control practices	159	97.0
Changing process of repeat prescription	90	54.9
Using e-script or health mail for prescriptions	151	92.1
Performing video consultations	159	97.0

**Fig. 1:**
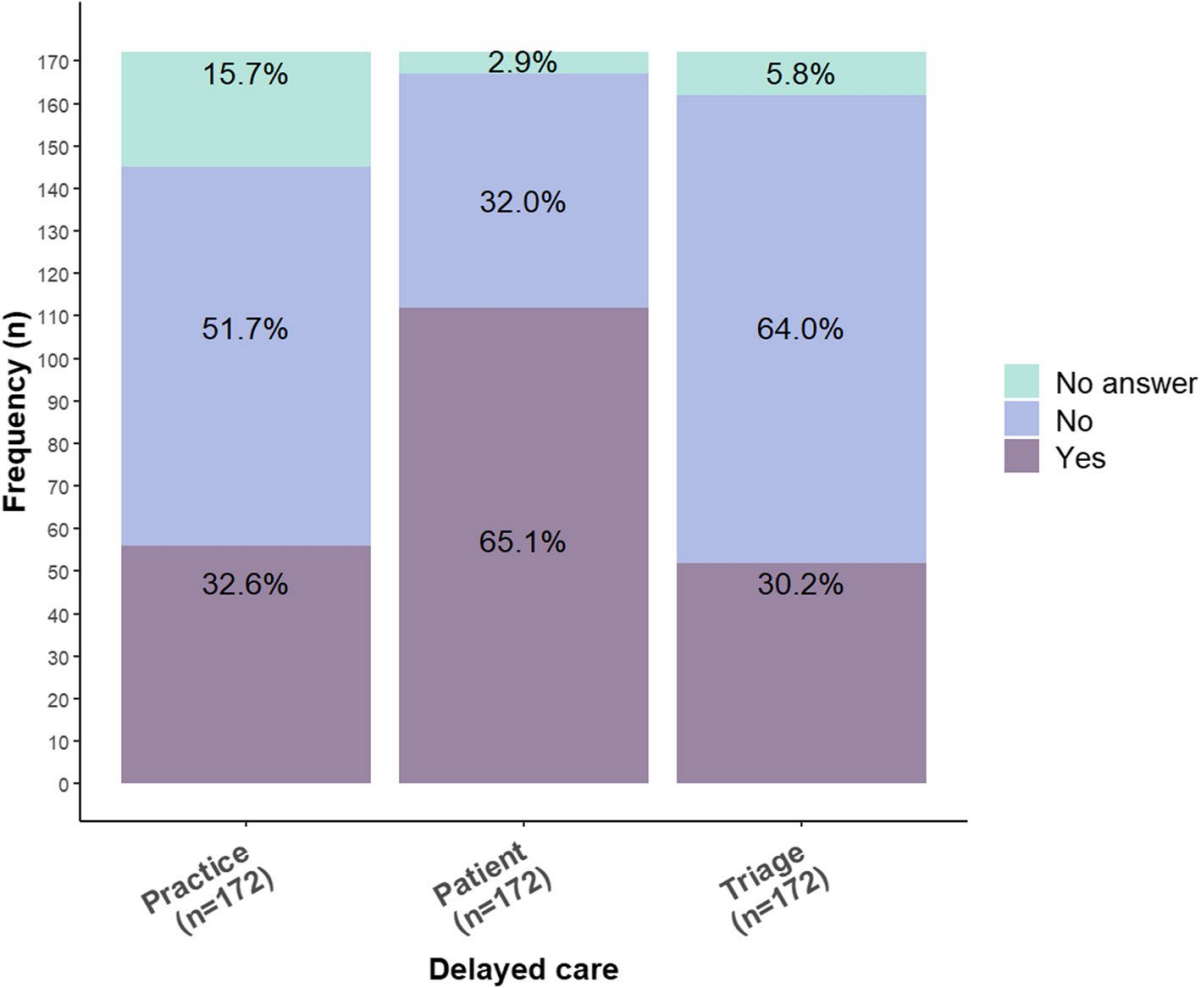
Reported occurrence for each potential PSI since the COVID-19 pandemic

Overall, 71% of practices (*n* = 122) reported at least one potential PSI and 16% (*n* = 28) reported all three potential PSI. Figure 1 shows the reported occurrence for each potential PSI (general practices could report more than one potential PSI). A total of 65% of practices reported they saw a patient late due to the patient’s lack of understanding of implemented processes (delayed/patient), 33% reported this delay due to the COVID-19 protocol mistakenly being followed for a non-COVID fever (delayed/practice), and 30% reported delays due to an urgent condition assessed incorrectly during triage procedure (delayed/triage). Some practices did not respond to any of the three potential scenarios of PSIs (delayed/practice (16%), delayed/patient (6%), or delayed/triage (3%)).

Univariate analyses showed an association with delayed/practice incidents between practices in the suburbs/small towns, number of GP trainees, having a practice manager, and having a nurse or care provider (Table 2). A higher number of paid staff was associated with reported delayed/patient incidents (OR 1.1 [CI 95% 1.0 to 1.2]) while small GP practices (one GP working) were less likely compared to large GP practices (four or more GPs working) to report having delayed/patient incidents. All PSIs were associated with changes in issuing repeat prescriptions (Table 2).

**Table 2:** Result of logistic regressions of factors associated with patient safety incidents in general practices in Ireland during the COVID-19 pandemic

	Delayed care/Practice OR (CI 95%)		Delayed care/Patient OR (CI 95%)		Delayed care/Triage OR (CI 95%)	
	Model I ^a^	Model II^b^	Model I ^a^	Model II^b^	Model I ^a^	Model II^b^
**Location of practice**						
Big (inner)city	Reference	Reference	Reference	Reference	Reference	Reference
Suburbs /(Small) town	3.01 (1.04 to 10.07)	4.19 (1.05 to 19.82)	0.85 (0.31 to 2.21)	0.86 (0.22 to 3.2)	1.02 (0.38 to 2.86)	0.79 (0.23 to 2.94)
Mixed urban–rural / Rural	1.47 (0.48 to 5.07)	1.88 (0.47 to 8.69)	1.21 (0.42 to 3.25)	1.11 (0.28 to 4.2)	0.52 (0.18 to 1.52)	0.32 (0.08 to 1.23)
**Number of people actively work**						
Paid staff	1.00 (0.95 to 1.06)	-	1.11 (1.04 to 1.19)	-	1.04 (0.99 to 1.10)	-
Unpaid staff	0.95 (0.46 to 1.84)	-	0.84 (0.44 to 1.67)	-	1.33 (0.67 to 2.57)	-
**Number of GPs**		-		-		-
4 or more	Reference	-	Reference	-	Reference	-
2 to 3	1.17 (0.56 to 2.42)	-	0.61 (0.28 to 1.3)	-	0.94 (0.45 to 1.94)	-
1	0.28 (0.07 to 0.84)	-	0.17 (0.06 to 0.44)	-	0.41 (0.12 to 1.16)	-
**Number of GPs trainees**		-		-		-
2 or more	Reference	-	Reference	-	Reference	-
1	1.28 (0.25 to 7.07)	-	0.73 (0.10 to 3.42)	-	0.45 (0.09 to 2.14)	-
0	0.65 (0.13 to 3.51)	-	0.46 (0.06 to 2.02)	-	0.43 (0.09 to 1.96)	-
**Practice manager working in practice**						
Yes	2.47 (1.06 to 6.29)	-	1.29 (0.61 to 2.65)	-	1.52 (0.71 to 3.45)	-
No	Reference		Reference		Reference	
**Nurse or care providers working in practice**						
Yes	3.29 (1.14 to 11.9)	-	4.43 (1.76 to 11.87)	-	2.66 (0.94 to 9.56)	-
No	Reference		Reference		Reference	
**Size of this practice (number of patients)**						
Under 2000	0.34 (0.08 to 1.16)	1.06 (0.17 to 5.6)	0.21 (0.06 to 0.61)	0.85 (0.19 to 3.65)	0.59 (0.16 to 1.94)	1.82 (0.35 to 9.07)
2000 to 3999	1.31 (0.51 to 3.41)	4.23 (1.15 to 17.06)	1.00 (0.38 to 2.63)	3.35 (0.95 to 12.93)	1.17 (0.45 to 3.07)	1.89 (0.55 to 6.89)
4000 to 5999	0.76 (0.26 to 2.15)	1.23 (0.34 to 4.57)	0.92 (0.33 to 2.53)	1.83 (0.53 to 6.74)	1.29 (0.47 to 3.58)	1.98 (0.58 to 7.15)
6000 to 7999	1.92 (0.60 to 6.31)	2.52 (0.61 to 11.02)	2.82 (0.75 to 13.7)	4.74 (1.05 to 27.61)	1.39 (0.44 to 4.35)	1.73 (0.44 to 6.90)
8000 and over	Reference	Reference	Reference	Reference	Reference	Reference
**Strategies implemented**						
Support telephone triage^c^						
Always	0.40 (0.11 to 1.36)	0.48 (0.11 to 1.99)	0.86 (0.22 to 2.82)	1.99 (0.39 to 9.24)	0.34 (0.10 to 1.09)	0.46 (0.11 to 1.85)
Never/Rarely/Sometimes/Mostly	Reference	Reference	Reference	Reference	Reference	Reference
Collaboration^d^						
Always	0.56 (0.26 to 1.19)	0.77 (0.29 to 1.91)	0.40 (0.19 to 0.83)	0.35 (0.14 to 0.87)	0.49 (0.21 to 1.05)	0.59 (0.23 to 1.43)
Never/Rarely/Sometimes/Usually	Reference	Reference	Reference	Reference	Reference	Reference
Measures guarding patient safety±						
Limiting the number of patients in waiting room	0.82 (0.33 to 2.07)	2.08 (0.53 to 8.59)	0.48 (0.16 to 1.22)	1.77 (0.33 to 9.85)	0.95 (0.38 to 2.51)	1.34 (0.37 to 4.96)
No longer use the waiting room	1.89 (0.94 to 3.83)	2.03 (0.63 to 6.66)	2.72 (1.31 to 5.94)	2.78 (0.81 to 11.14)	1.67 (0.84 to 3.35)	2.32 (0.79 to 6.79)
Structural changes to the reception area	3.80 (1.60 to 10.15)	3.46 (1.19 to 11.4)	2.77 (1.31 to 5.89)	1.64 (0.60 to 4.49)	1.84 (0.82 to 4.47)	1.25 (0.46 to 3.63)
Changing process of repeat prescription	2.26 (1.12 to 4.65)	2.41 (0.87 to 6.99)	3.78 (1.90 to 7.71)	6.71 (2.54 to 19.6)	2.17 (1.09 to 4.46)	2.27 (0.89 to 6.08)
Using e-script or health mail for prescriptions	0.90 (0.27 to 3.21)	1.05 (0.25 to 4.63)	1.27 (0.36 to 4.03)	0.67 (0.16 to 2.68)	0.75 (0.23 to 2.60)	0.59 (0.15 to 2.43)

^a^Model I: Univariate logistic regressions

^b^Model II: Multivariate logistic regressions

^c^Support telephone triage: In the situation where, telephone triage is performed by someone other than a GP in this practice and he/she needs support when assessing a call, he/she can rely on support from a GP

^d^Collaboration: If an incident about quality of care occurs in the practice, this is discussed at a(n) (online) team meeting (either with the whole team or only with the health professionals)

±Performing triage before patients entering this practice, performing telephone triage, increasing infection control practices, and performing video consultations were not included in these models because they had few data in category “Unchecked/No”

After assessing multicollinearity and confounding, location and size of the practice were included as independent practice variables in the multivariable logistic regression. Structural changes to the reception area during the COVID-19 was significantly associated with delayed care due to practice factors. Suburbs/small towns and a practice size of 2,000 to 3,999 patients, were associated with delayed/practice incidents, but no linear association was observed with other categories of practice size (Table 2). Changes in the process of repeat prescriptions was associated with delayed care due to patient factors. Delay due to triage was not associated with any practice variables.

As the practice size showed a moderate correlation with other practice characteristics, a second multivariable model was run excluding this variable and including staff variables (number of GP, number of GP trainees, having a practice manager, and having a nurse or care provider), see additional file 1. This model showed that delayed/triage outcome was less likely in mixed urban-rural/rural locations compared to large cities (OR 0.2 [CI 95% 0.04 to 0.7]) but more likely when the practice no longer used their waiting room (OR 3.0 [CI 95% 1.0 to 9.3]).

## Discussion

This study showed some of the challenges encountered in general practice in Ireland at the start of the COVID-19 pandemic and provides context to understand the effect and emphasises the health system’s resilience to guarantee high-quality care and patient safety.

Delayed care due to patient factors (65%) were most often due to patients’ misunderstanding of changing engagement or provision of care of the practice. Fournier et al. found similar frequency reports by French GPs (from March to June 2020) related to the patient (delayed attending or did not seek consultation because of the fear of contracting COVID-19) [10]. A nationwide survey of more than 150,000 participants in Ireland at the start of the pandemic, showed that many people delayed care or contacting their GP, generally because of anxiousness and concern about catching the virus (70%) [20]. PSIs due to patient factors are however difficult for GPs to assess as they may not be aware of these, in particular if the delay resulted in resolution of the issue.

Additionally, compared with some reports before the pandemic, the frequency of incidents did not differ widely [17, 18, 21]. For example, a French cross-sectional survey of primary care in 2013 by Chaneliere et al. reported 428 PSIs; 13% were due to human factors related to patients. In another study in 2013, French GPs reported that 76% of PSIs were associated with workflow in the practice and communication between providers and patients [18], and a 2016 English/Australian report showed similar PSIs related to delays in patients accessing a GP (24%) and errors in information communication (7%) [21].

Delayed care due to practice factors (33%) was higher in suburban and small-town practices and practices where structural changes were made to the reception area. No consistent association with a larger or smaller patient population was observed. The lack of consistent patterns, shows the adaptability and flexibility of general practice under enormous strain. The only exception is for repeat prescriptions, where the flexibility of the primary care system resulted in sustainable positive changes in the implementation of electronic prescribing and improved collaboration between pharmacies and GPs [22]. Overall, as was found in other studies, general practice did benefit from autonomy in deciding how they want to work during health emergencies [23].

Since the COVID-19 pandemic, telemedicine and digital health have shown their potential benefits and contribution for health services. At the start of the pandemic, between 80% and 97% of consultations in general practice were face-to-face [24]. In the United Kingdom, before the pandemic, 30% of the consultations were over the phone, which suddenly increased to nearly 85% of the consultations during the pandemic [24, 25]. Ireland saw telephone consultations increase to 57% (from 10% previously), while according to a point prevalence study [12], telemedicine changed from 0 to 82% (April 2020) and 75% (February 2021) over COVID but has stabilised to 56% in 2022 again [26]. This allowed GPs to maintain contact with patients at a time when there was little understanding of the risk factors or infection patterns of COVID-19 [23, 27]. Since general practice has returned to face-to-face consultations again, even in a hybrid format, online/telephone consultations have found a place in general practice [26]. However, different factors which influence online or telephone consultations should be considered, such as the type of the health system, lack of technical access, contexts, cultures, lack of skills (i.e. older people), psychologically challenged individuals and geographic areas [28, 29].

This study has several limitations related to the time of data collection (start of the COVID-19 pandemic), use of self-reported questionnaires, inclusion of a self-selected sample and that the number of incidents was not collected [14, 15]. For instance, this study did not capture or measure some negative consequences in primary care during the pandemic (because it was carried out at the early stage of the pandemic), such as the follow-up of people with chronic illnesses. It has been observed this population group had significant barriers to diagnosis, treatment and follow-up visits and maybe even dropped out of care [30]. However, its strength lies in the recording of changes during the pandemic which provides a context for research set during this time in general practice and an understanding how changes may or may not have impacted health care. Despite the low response in this analysis (12%), the number of general practices included is representative of Irish general practices. Compared to other countries included in the PRICOV-19 study, the response in Ireland was higher than Sweden, Poland, Norway, Malta, Latvia, France, Denmark and Bosnia and Herzegovina [15]. Also, other studies in similar groups observe a response similar or lower than this when using online survey modes (about 10% less compared to other modes) [31, 32]. Furthermore, the measurement of these potential patient safety outcomes is too limited to make meaningful comparative statements due to using self-reported questionnaires based on five binary questions. Capturing the entire concept would require a validated scale for future research, such as the multi-dimensional patient safety instrument for primary care developed by Ricci-Cabello I et al. in 2016. This instrument includes five domains to cover patients’ experiences and perceptions of patient safety: (1) what the practice does to create a safe environment (practice activation), (2) how proactive the patient is concerning their safety (patient activation), (3) patient safety events experiences, (4) outcomes of patient safety events (harm) and (5) how safe patients perceive their practice to be (overall patient safety perceptions) [33].

## Conclusions

With the COVID-19 pandemic, dramatic changes were observed in the delivery of care through general practices in Ireland. Few factors were associated with reported occurrence of PSIs and were generally non-consistent. Delayed care due to patient factors was associated with issues with delayed prescriptions and sustained improvements were seen early on to circumvent this. The lack of consistent patterns, potentially confirms that the autonomous decisions made in general practice in response to the challenges of the COVID-19 pandemic could have benefitted patient safety.

### Supplementary Information

Additional file 1: Multivariable logistic regressions including staff composition variables (GPs, GPs trainees, practice manager, and nurse or care provider).

## Data Availability

The datasets generated and/or analysed during the current study are not publicly available due to the legal arrangements with the overall PRICOV-19 study PI’s institution but are available from the corresponding author on reasonable request with the appropriate approvals.

## Abbreviations

PSI: Patient safety incident
GP: General practitioner
ICGP: Irish College of General Practitioners
VIF: Variance inflation factor
OR: Odds ratio
CI: Confidence interval
