# Supplementary material for: Patient safety incidents in Irish general practice during the COVID-19 pandemic: an exploratory practice level analysis
Source: BMC Prim Care. 2024 May 29;24(Suppl 1):288. doi: 10.1186/s12875-024-02439-9 (PMC11137878; doi:10.1186/s12875-024-02439-9)
Supplement: Supplementary file 1 — Additional file 1: Multivariable logistic regressions including staff composition variables (GPs, GPs trainees, practice manager, and nurse or care provider). [file 12875_2024_2439_MOESM1_ESM.docx]

Additional file 1. Multivariable logistic regressions including staff composition variables (GPs, GPs trainees, practice manager, and nurse or care provider).

|  | **Delayed care/Practice  OR (CI 95%)** | **Delayed care/Patient**  **OR (CI 95%)** | **Delayed care/Triage**  **OR (CI 95%)** |
| --- | --- | --- | --- |
| **Location of practice** |  |  |  |
| Big (inner)city | Reference | Reference | Reference |
| Suburbs /(Small) town | 3.63 (0.94 to 16.57) | 0.62 (0.15 to 2.35) | 0.55 (0.14 to 2.13) |
| Mixed urban–rural / Rural | 1.17 (0.28 to 5.43) | 0.76 (0.17 to 3.1) | 0.17 (0.04 to 0.72) |
| **Number of GPs** |  |  |  |
| 4 or more | Reference | Reference | Reference |
| 2 to 3 | 1.88 (0.71 to 5.25) | 0.59 (0.22 to 1.58) | 1 (0.38 to 2.68) |
| 1 | 1.11 (0.2 to 5.69) | 0.46 (0.1 to 2.07) | 2 (0.43 to 9.28) |
| **Number of GPs trainees** |  |  |  |
| 2 or more | Reference | Reference | Reference |
| 1 | 1.41 (0.2 to 10.12) | 1.06 (0.11 to 7.19) | 0.69 (0.1 to 4.91) |
| 0 | 1.07 (0.16 to 7.71) | 1.16 (0.12 to 8.08) | 0.93 (0.14 to 6.65) |
| **Practice manager working in practice** |  |  |  |
| Yes | 1.52 (0.48 to 5.19) | 0.33 (0.09 to 1.01) | 1.55 (0.51 to 5.06) |
| No | Reference | Reference | Reference |
| **Nurse or care providers working in practice** |  |  |  |
| Nurse or care providers | 1.53 (0.32 to 7.89) | 2.55 (0.59 to 11.63) | 4.57 (1.00 to 26.37) |
| No | Reference | Reference | Reference |
| **Strategies implemented** |  |  |  |
| Support telephone triage^¶^ |  |  |  |
| Always | 0.57 (0.12 to 2.51) | 1.86 (0.38 to 8.33) | 0.51 (0.12 to 2.33) |
| Never/Rarely/Sometimes/Mostly | Reference | Reference | Reference |
| Collaboration^¶¶^ |  |  |  |
| Always | 0.76 (0.28 to 1.96) | 0.41 (0.16 to 1.04) | 0.6 (0.22 to 1.55) |
| Never/Rarely/Sometimes/Usually | Reference | Reference | Reference |
| Measures guarding patient safety± |  |  |  |
| Limiting the number of patients in waiting room | 1.64 (0.41 to 6.81) | 1.22 (0.23 to 6.60) | 1.57 (0.40 to 6.34) |
| No longer use the waiting room | 1.69 (0.55 to 5.24) | 1.88 (0.53 to 7.62) | 3.03 (1.01 to 9.28) |
| Structural changes to the reception area | 3.41 (1.15 to 11.32) | 1.92 (0.69 to 5.37) | 0.9 (0.31 to 2.72) |
| Changing process of repeat prescription | 1.65 (0.63 to 4.34) | 5.8 (2.26 to 16.17) | 2 (0.79 to 5.22) |
| Using e-script or health mail for prescriptions | 1.39 (0.29 to 7.43) | 0.84 (0.19 to 3.46) | 0.64 (0.15 to 3.16) |
| ¶Support telephonic triage: In the situation where, telephonic triage is performed by someone other than a GP in this practice and he/she needs support when assessing a call, he/she can rely on support from a GP  ¶¶Collaboration: If an incident about quality of care occurs in the practice, this is discussed at a(n) (online) team meeting (either with the whole team or only with the health professionals)  ±Performing triage before patients entering this practice, performing telephone triage, increasing infection control practices, and performing video consultations were not included in these models because they had few data in category “Unchecked/No” | | | |
